# Mesenchymal Stem Cells Enhance Therapeutic Effect and Prevent Adverse Gastrointestinal Reaction of Methotrexate Treatment in Collagen-Induced Arthritis

**DOI:** 10.1155/2021/8850820

**Published:** 2021-01-09

**Authors:** Qiming Zhai, Jiayi Dong, Xuesi Zhang, Xiaoning He, Dongdong Fei, Yan Jin, Bei Li, Fang Jin

**Affiliations:** ^1^State Key Laboratory of Military Stomatology & National Clinical Research Center for Oral Diseases & Shaanxi International Joint Research Center for Oral Diseases, Center for Tissue Engineering, School of Stomatology, The Fourth Military Medical University, Xi'an, Shaanxi 710032, China; ^2^Department of Orthodontics, School of Stomatology, Fourth Military Medical University, Xi'an, Shaanxi 710032, China; ^3^Department of Aerospace Medicine, Fourth Military Medical University, Xi'an, Shaanxi 710032, China

## Abstract

Rheumatoid arthritis (RA) is a systemic autoimmune disease characterized by articular destruction and functional loss. Methotrexate (MTX) is effective in RA treatment. However, MTX induces several adverse events and 20%-30% of patients do not respond to MTX. Thus, it is urgent to enhance the therapeutic effects and reduce the side effects of MTX. Recent studies showed that mesenchymal stem cells (MSCs) were participants in anti-inflammation, immunoregulation, and tissue regeneration. However, whether the combined application of MSCs and MTX promotes the therapeutic effects and reduces the side effects of MTX has not been studied. In this study, we used bovine type II collagen to induce rheumatoid arthritis in mice (collagen-induced arthritis, CIA). Then, CIA mice were subjected to MTX or MSC treatment, or both. The therapeutic effect and adverse events of different treatments on RA were evaluated with micro-CT, HE staining, and immunohistochemistry in vivo. Apoptosis and proliferation of MODE-K cells were measured after treated with MTX or/and cocultured with UCs. To test M2 polarization, Raw264.7 macrophages were stimulated by MTX with different concentrations or cocultured with UCs. We found that the combined application of MSCs and MTX increased the therapeutic effects on RA, as evidenced by decreased arthritis score, inflammatory responses, and mortality. Moreover, in this combination remedy, MTX prefers to suppress inflammation by facilitating macrophage polarization to M2 type while UCs prefer to eliminate gastrointestinal side effects of MTX via mitigating the apoptosis of intestinal epithelial cells. Thus, a combination of MTX and UCs is a promising strategy for RA treatment.

## 1. Introduction

Rheumatoid arthritis (RA) is a systemic autoimmune disease, characterized by nonsuppurative inflammation of joints and joint tissues [1]. It is mainly manifested as joint synovitis, which eventually leads to joint cartilage, ligament, and tendon damage. Methotrexate (MTX) is an important disease-modifying antirheumatic agent (DMARD), which serves its function on suppressing the division and proliferation activity of lymphocytes in RA [2, 3]. For its superior efficacy for the treatment of RA in many clinical and basic research, MTX has been considered as the initial therapy for RA [2]. According to the latest updated European League Against Rheumatism (EULAR) rheumatoid arthritis (RA) management recommendations in 2019 and the American College of Rheumatology (ACR) Guideline for the treatment of RA in 2015, therapy with DMARDs should be started as soon as the diagnosis of RA is made and MTX should be part of the first treatment [4, 5].

However, it is worth noting that MTX induces gastrointestinal side effects, kidney damage, bone marrow suppression, pulmonary toxicity, and psychological disorders [3, 6–8], which limits the application of MTX in clinic. Besides, a large number of individuals do not respond to these drugs adequately [9]. Therefore, a new therapeutic strategy is required to enhance the therapeutic effects and reduces the side effects of MTX.

Advancements in stem cell engineering and regenerative medicine therapies provide potential strategies for the treatment of RA [10, 11]. Mesenchymal stem cells (MSCs) showed outstanding immunomodulatory and anti-inflammatory ability in RA [11, 12]. Previous studies have revealed that systemic infusion of MSCs alleviates the symptoms of RA on collagen-induced arthritis (CIA) mice, and umbilical cord-derived MSCs (UCs) exert the best therapeutic effect [13]. Over the past decades, multiple studies have focused on the therapeutic effect of MSCs on attenuating RA symptoms. Meanwhile, it is notable that MSCs can promote tissue repair and regeneration in a series of diseases as well, such as colitis, liver injuries, and kidney injuries [12, 14]. However, MSCs are far from clinic use for the dispute on administration dosage and the unsatisfactory therapeutic effect on acute symptoms of RA. Under this circumstance, we proposed that combined application of UCs and MTX might enhance the curative effect and mitigate the adverse side effects of MTX.

In the present study, collagen-induced arthritis (CIA) mice were administered with MTX and UCs separately or simultaneously. Our data provide evidence for the complementary effects of UCs and MTX, which suggest that combined application of MTX and UCs is more effective and safer for the treatment of RA and highlights the significance of stem cell therapy.

## 2. Materials and Methods

### 2.1. Animal Studies

DBA1/J mice (female, 6-8 weeks old) were obtained from Beijing Vital River Laboratory Animal Technology Company and housed in pathogen-free conditions with constant temperature, humidity, and 12 : 12-hour light-dark cycle. Food and water were provided ad libitum. All animal experiments were performed according to the guidelines set by the Animal Care Committee of the Fourth Military Medical University, Xi'an, China.

Collagen-induced arthritis (CIA) was induced as previously described [13]. Briefly,100 *μ*g bovine type II collagen (CII) (Chondrex, Redmond, WA, USA) was emulsified with an equal volume of Freund's complete adjuvant and injected into the base tail of DBA1/J mice for 2 weeks. Freund's incomplete adjuvant was injected for the later 2 weeks. The swollen claws of mice represent a symptom of the CIA model. Forty CIA mice were divided into four experiment groups: (1) CIA group: CIA mice were injected with PBS as a control group, (2) MTX-treated group: CIA mice were injected intraperitoneally with MTX (1 mg/kg) every two days for 8 weeks, (3) UC-treated group: CIA mice were injected intravenous with 1 × 10^6^ UC, and (4) MTX+UC-treated group: CIA mice were injected intraperitoneally with MTX (1 mg/kg) every two days for 8 weeks. Meanwhile, a single dose of 1 × 10^6^ UCs was intravenously injected into CIA mice after MTX treatment for 1 week. All mice were euthanatized after MTX or UC or MTX+UC treatment for 8 weeks.

### 2.2. Cell Culture

Mesenchymal stem cells (MSCs) were obtained from human umbilical cords after harvesting informed consent for research purposes, which was approved by the Ethics Committee (Institutional Review Board for Human Subjects Research) of the School of Stomatology, Fourth Military Medical University (FMMU). The umbilical cords were washed with phosphate-buffered saline (PBS) and cut into 1 mm^3^ tissue blocks after removing blood vessels. Then, the tissue blocks were plated on a polystyrene tissue culture flask and suspended in low-glucose Dulbecco's modified Eagle's medium (L-DMEM) (Hyclone, Logan, Utah, USA) with 10% fetal bovine serum (FBS, Hyclone, Logan, Utah, USA), 100 U/mL penicillin (Invitrogen, Carlsbad, CA, USA), and 100 mg/mL streptomycin (Invitrogen, Carlsbad, CA, USA) for 7 days. Primary cells were expanded in a 37°C humidified environment with 5% CO_2_ and 95% air. The fourth passage cells were used for experiment in the present study. MODE-K cells were treated with 20 ng/mL MTX or/and cocultured with UCs (1 × 10^6^) for 24 h and used for measuring apoptosis and proliferation. To measure M2 polarization, primary macrophages were isolated from the peritoneal cavity of CIA mice. Raw264.7 macrophages were stimulated by MTX with different concentrations of 0, 0.01, 0.05, 0.1, and 0.5 *μ*g/mL or cocultured with UCs (1 × 10^6^) for 24 h. T cells were sorted from spleens of CIA mice. Briefly, after grinding spleens, strained cells were rinsed with PBS and then resuspended in ACK lysis buffer (2 mL/spleen) (Beyotime Biotechnology, China), at room temperature for 10 min. Centrifuged pellet cells were resuspended in T cell medium with CD28 antibody at 2 *μ*g/mL and seeded on CD3-coated dishes.

### 2.3. Flow Cytometry Assay

5 × 10^5^ cells were incubated with anti-human CD34, CD45, CD90, and CD105 antibodies labelled with PE and anti-human CD73 antibodies labelled with FITC (all from eBioscience, San Diego, CA, USA) for 30 min, respectively, at room temperature. Primary macrophages or Raw264.7 cells were stained with CD206 labelled with PE for 30 min at room temperature. T cells were stained with CD4, CD8, CD25, and Foxp3. All the surface markers were analyzed by flow cytometry with a Beckman Coulter Epics XL cytometer (Beckman Coulter, USA). Apoptosis was determined by Annexin V and PI (Roche) staining according to the manufacturer's protocol. Proliferation was determined by the EdU Assay Kit (Beyotime Biotechnology, China).

### 2.4. Osteogenic Differentiation Assay

UCs were seeded in six-well plates with a density of 5 × 10^5^ and cultured in a basal medium for 24 h. Then, cells were cultured in an osteogenic medium containing 10% FBS, 10 mM *β*-glycerophosphate (Sigma-Aldrich, St. Louis, MO, USA), 100 nM dexamethasone (Sigma-Aldrich, St. Louis, MO, USA), and 50 *μ*g/mL ascorbic acid (Sigma-Aldrich, St. Louis, MO, USA) for 28 days. The medium was refreshed every 3 days. The cells were washed twice by PBS and fixed in 60% isopropanol for 1 min (Sigma-Aldrich, St. Louis, MO, USA). Alizarin red (Sigma-Aldrich) staining was performed, and photographs were taken by an inverted microscope (Olympus, Tokyo, Japan).

### 2.5. Adipogenic Differentiation Assay

UCs were cultured with an adipogenic-inducing medium containing 0.5 mM isobutylmethylxanthine (MP Biomedicals, Santa Ana, CA, USA), 0.5 mM dexamethasone (MP Biomedicals, Santa Ana, CA, USA), 60 mM indomethacin (MP Biomedicals, Santa Ana, CA, USA), and 10 mg/mL insulin. The medium was refreshed every 3 days. The cells were washed twice by PBS and fixed in 4% paraformaldehyde (Sigma-Aldrich, St. Louis, MO, USA). Then, Oil red O (Sigma-Aldrich, St. Louis, MO, USA) staining was performed and photographs were taken by an inverted microscope (Olympus, Tokyo, Japan).

### 2.6. MTT Assay

A total of 2 × 10^3^ UCs was plated in 96-well culture plates with a basal medium or supplied with different MTX concentrations (0, 1, 10, and 100 *μ*g/mL) for 24 hours. 3-(4,5-dimethylthiazol-2-yl)-2,5-diphenyltetrazolium bromide (MTT) assay was performed according to the manufacturer's instructions. Briefly, 20 *μ*L MTT solution (Sigma-Aldrich, 5 mg/mL) was incubated with UCs for 4 hours. 150 *μ*L dimethylsulfoxide (DMSO, Sigma-Aldrich) was used to dissolve formazan salts after the medium was discarded. The cell viability was analyzed by a microplate reader (ELx800, BioTek Instruments Inc., Highland Park, USA) at 490 nm.

### 2.7. Histological and Immunohistochemical Staining

Hind limbs were harvested and fixed in 4% paraformaldehyde at 4°C for 48 h. After that, the tissues were decalcified in 17% EDTA for 15 days, embedded in paraffin, and 8 *μ*m thick serial sections were performed for haematoxylin-eosin staining. Sections of the liver, lung, and kidney were performed as the same processes. Immunohistochemical staining was performed with previously described procedures [15]. Briefly, paraffin sections were incubated with primary antibodies IL-1*β* (1 : 200; Abcam, Cambridge, UK) and TNF-*α* (1 : 200; Abcam, Cambridge, UK) overnight at 4°C. After rinsing with PBS three times, sections were incubated with secondary antibodies (1 : 1000), which were purchased from Vector Laboratories. A light microscope (DM6B; Leica Microsystems, Heerbrugg, Switzerland) was used to observe stained sections, and the photographs were evaluated by ImageJ (Media Cybernetics, Maryland, USA) from three randomly selected views of each specimen.

### 2.8. ELISA

Serum was harvested from mouse blood after centrifuging at 3000 rpm for 20 min. Enzyme-Linked Immunosorbent Assay (ELISA) kits (Yanhui Biotechnology, Shanghai, China) were used to analyze the level of IL-1*β*, RF, and TNF-*α* in serum according to the manufacturer's instructions.

### 2.9. Micro-CT

Micro-CT (eXplore Locus SP, GE Healthcare) was used to scan mouse hind limbs as previously described [13]. The scanning parameters are settled as follows: 14 *μ*m resolution, 360° rotation angle, 80 kV, 80 *μ*A, 3000 ms exposure time, and 2 × 2 pixel combination. 3D reconstruction was built to analyze the paw bone with Micview V2.1.2 and ABA.

### 2.10. Disease Severity Score

Arthritis was evaluated by using a scoring system as previously described [16]. Each hind paw was individually evaluated every week after MTX or UC treatment. The disease severity was scored on a scale of 1-4: none symptoms scored 0; mild swelling confined to the tarsals or ankle joint scored 1; mild swelling extending from the ankle to the tarsal bones scored 2; moderate swelling extending from the ankle to the metatarsal joints scored 3; and severe swelling encompassing the ankle, foot and digits, or ankylosis of the limb scored 4. Scores of both hind paws were added up to a total score to represent severity.

## 3. Results

### 3.1. Isolation and Characterization of MSCs with or without MTX Treatment

MSCs were successfully isolated from umbilical cords (UCs). The cells exhibited positive expression of MSC surface markers (CD90, CD73, and CD105) and negative expression of hematopoietic markers (CD34 and CD45). Meanwhile, they maintained this characteristic pattern of MSCs after being stimulated by MTX (Figure S1a). We also cultured UCs in an osteogenic differentiation medium or adipogenic differentiation medium to detect their differentiation potential. Alizarin red staining (Figure S1b) and Oil Red staining (Figure S1c) showed that mineralized nodules and lipid droplet formation of UCs could be induced to the same extent both treated with and without MTX. MTT assay was conducted to illustrate that the proliferation of UCs was not affected by MTX with different concentrations from 0 to 100 *μ*g/mL (Figure S1d). Additionally, apoptosis of UCs was always kept at a low level below 2% even after being stimulated by MTX at the highest dose of 100 *μ*g/mL (Figure S1e). Based on these results, the isolated UCs meet the International Society for Cellular Therapy (ISCT) criteria of MSCs [17] and MTX has not impaired the differentiation potential and growth of UCs.

### 3.2. Combined Use of UCs and MTX Enhanced the Therapeutic Efficacy of MTX

**Figure 1:**
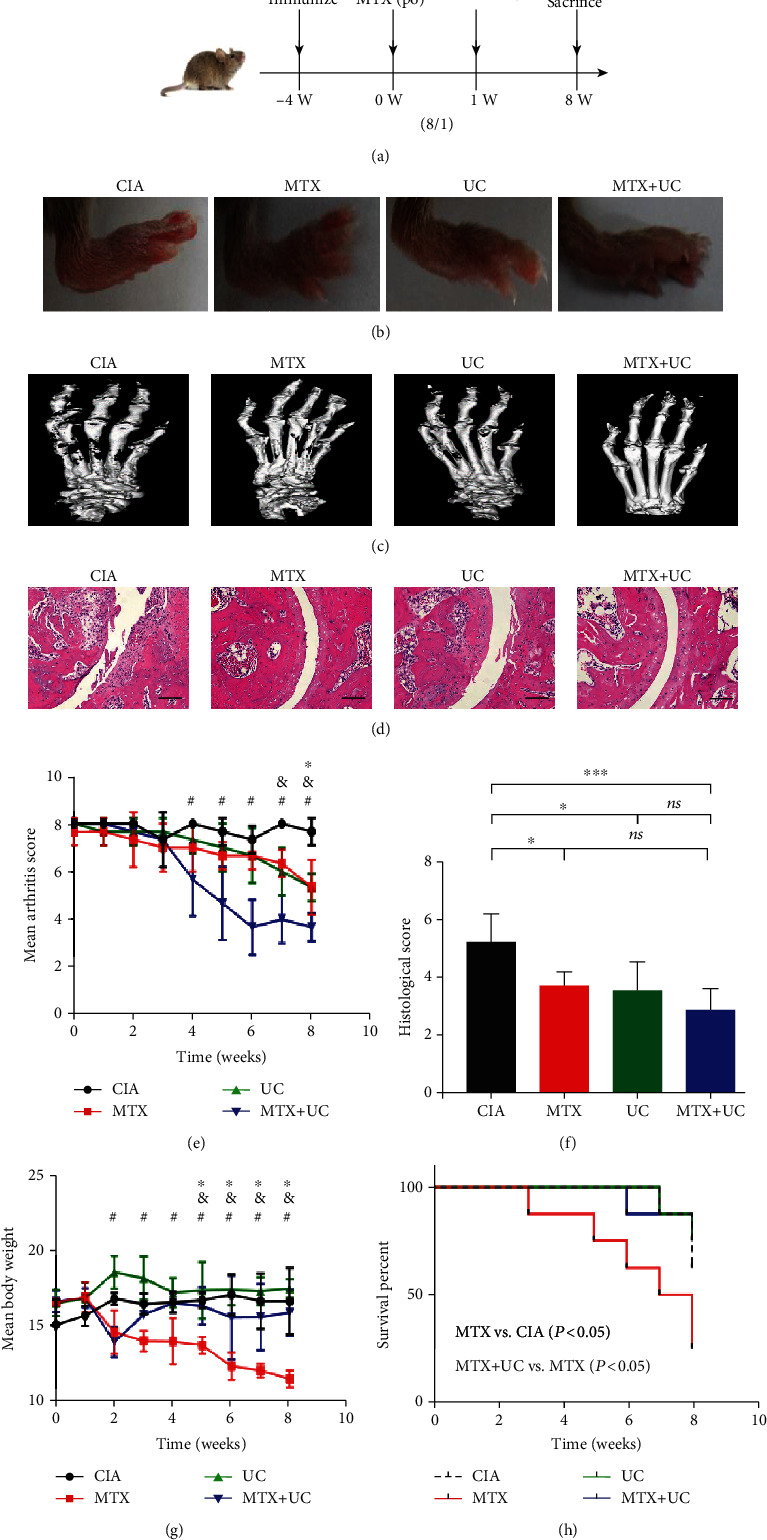
Combined use of UCs and MTX enhanced the therapeutic efficacy of MTX. (a) The therapeutic design of the CIA mice. (b, c) Combined use of MTX and UCs showed the mildest paw swelling and bone erosion. (d) H&E staining showed that the MTX+UC group had the best effect on suppressing synovitis, erosion, and inflammatory cell infiltration compared to the other three groups. (e) Mean arthritis score of four groups was evaluated every week. ^∗^*P* < 0.05, MTX vs. CIA; ^&^*P* < 0.05, UC vs. CIA; ^#^*P* < 0.05, MTX+UC vs. CIA. (f) Histological score evaluation in four groups. ^∗^*P* < 0.05; ^∗∗∗^*P* < 0.001; ns: no significance (*P* > 0.05). (g) Combination of MTX and UCs protected CIA mice from severe weight loss caused by MTX. ^∗^*P* < 0.05, MTX vs. CIA; ^&^*P* < 0.05, MTX+UC vs. MTX; ^#^*P* < 0.05, UC vs. MTX. (h) Combination of MTX and UCs prolonged the lifespan of the mice treated by MTX. Scale bar, 250 *μ*m. *n* = 3‐10 per group. Data are shown as mean ± SD.

To evaluate the efficacy of the combined application of MTX and UCs on RA, CIA mice were treated with MTX and UCs separately or simultaneously for 8 weeks (Figure 1(a)). After 8 weeks, the joint inflammation and injury in CIA mice were evaluated by gross observation, micro-CT analysis, and histological analysis. As shown in Figure 1, both MTX and UC treatment alone relieved the paw swelling (Figure 1(b)) and bone erosion at the joints (Figure 1(c)), and combined use of MTX and UCs showed the mildest bone erosion. In addition, the combined use of MTX and UCs showed the best effect on reducing the inflammatory infiltration and improving the integrity of bone surface in local joints (Figure 1(d)). Arthritis score and histological score were used to measure the severity of RA; the results showed that combined application of MTX and UCs showed a lower severity score (Figures 1(e) and 1(f)).

To assess the safety of alone or combined use of MTX and UCs, body weight and survival rate were recorded weekly. MTX treatment alone led to a significant decrease in body weight of CIA mice; however, MTX and UC combination blunted the adverse effect of MTX alone (Figure 1(g)). More importantly, the combination of MTX and UCs prolonged the lifespan of CIA mice compared to MTX alone treatment, which was indicated by the survival rate (Figure 1(h)). In summary, those results suggested that combined application of MTX and UCs enhanced the therapeutic effect and safety comparing to MTX treatment alone.

### 3.3. Combined Use of UCs and MTX Reduced the Levels of Inflammatory Cytokines and Rheumatoid Factors

**Figure 2:**
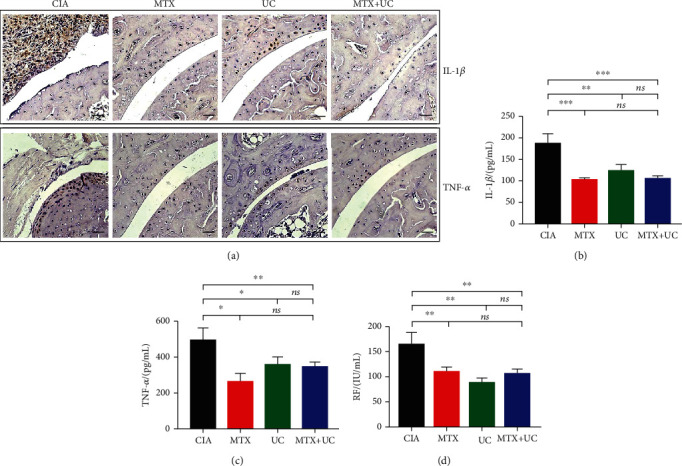
Combined use of UCs and MTX reduced the levels of inflammatory cytokines and rheumatoid factors. (a) Representative images of immunohistochemical staining in joints of CIA mice. The MTX+UC group reduced the expression of IL-1*β* and TNF-*α* accumulated in joints compared to the other three groups. Serum levels of TNF-*α* (b), IL-1*β* (c), and rheumatoid factor (d) were measured by ELISA. Scale bar, 100 *μ*m. *n* = 3 per group. Data are shown as mean ± SD. ^∗^*P* < 0.05; ^∗∗^*P* < 0.01; ^∗∗∗^*P* < 0.001; ns: not significant (*P* > 0.05).

TNF-*α*, IL-1*β*, and rheumatoid factor (RF) contribute to the pathological progress of rheumatoid. Immunohistochemical staining was performed to evaluate the effect of the combination of MTX and UCs on inflammatory cytokine accumulation in joint tissue. As shown in Figure 2(a), UCs or MTX treatment alone reduced the TNF-*α* and IL-1*β* accumulation in the joint. Moreover, the combination of MTX and UCs furtherly enhanced this effect. However, compared with MTX or UC alone, comminated application of MTX and UCs cannot further reduce the concentration of IL-1*β*, TNF-*α*, and RF in serum (Figures 2(b)–2(d)). Those data indicated that the combined use of MTX and UC only exerted a synergistic effect in blocking inflammation responses in joint cavities, which might explain the further improved symptom in CIA mice receiving MTX alone.

### 3.4. Combination of UCs and MTX Ameliorated Side Effects of MTX

**Figure 3:**
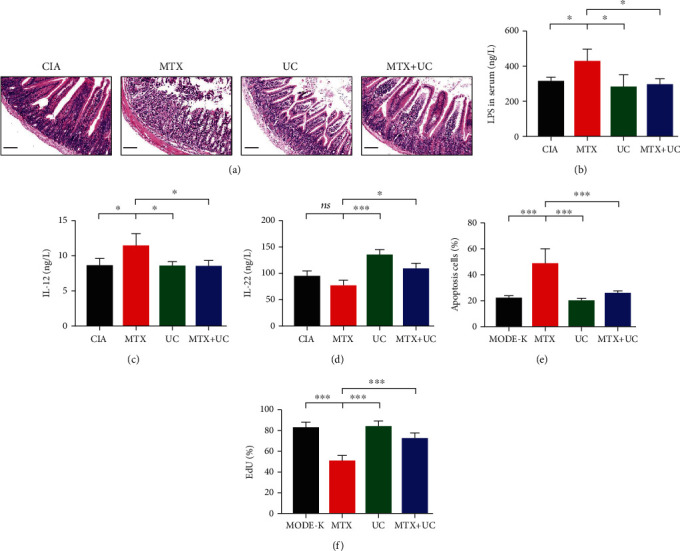
Combination of UCs and MTX ameliorated side effects of MTX. (a) Representative images of H&E staining of intestinal sections of four groups. Combined application of MTX and UCs to CIA mice downregulated the content of LPS (b) and IL-12 (c) in serum compared to CIA mice treated by MTX. (d) Using UCs or combination of MTX and UCs to treat CIA mice promoted the expression of IL-22 in serum compared to CIA mice treated by MTX. (e) Apoptosis of MODE-K treated by MTX and cocultured with UCs was analyzed by flow cytometry. (f) Proliferation of MODE-K treated by MTX and cultured with UCs was assessed by EdU analysis. Scale bar, 100 *μ*m. *n* = 4‐6 per group. Data are shown as mean ± SD. ^∗^*P* < 0.05; ^∗∗^*P* < 0.01; ^∗∗∗^*P* < 0.001; ns: not significant (*P* > 0.05).

It has been reported that MTX exerted different probability of side effects in varying systems [18], such as hepatotoxicity, pulmonary toxicity, and gastrointestinal side effects. We next investigated whether the combination of UCs and MTX alleviated the adverse effects of MTX alone. MTX treatment alone led to intestinal epithelial structure disorder and fuzzy small intestinal villi (Figure 3(a)). However, combined use with UCs significantly alleviated the intestinal epithelial injury (Figure 3(a)). In addition, MTX alone increased the LPS and IL-12 concentration in serum, suggesting destroyed intestinal epithelium barrier and activated enteritidis (Figures 3(b) and 3(c)). However, the combined application of MTX and UCs significantly suppressed the LPS and IL-12 induced by MTX (Figures 3(b) and 3(c)). On the other hand, the combined use of MTX and UCs rescued the decreased expression of IL-22, promoting intestinal injury healing [19], in mice receiving MTX treatment (Figure 3(d)).

To further investigate the effect of UCs on the intestinal system, we exposed the murine intestinal epithelial cell line (MODE-K) to either MTX or UC treatment, or both. It is observed that MTX significantly activated the apoptosis and suppressed the proliferation of MODE-K, and this effect was markedly blunted by MTX and UC cotreatment (Figures 3(e) and 3(f)). Moreover, we also observed that MTX destroyed the organized architecture of the lungs, livers, and kidneys. And those adverse effects were mitigated by MTX and UC combination (Figure S2). Overall, our results showed that combination use with UCs alleviated the adverse side effects of MTX alone in CIA mice.

### 3.5. Combination Medication Promoted Macrophage Polarization and Enhanced the Population of Tregs

**Figure 4:**
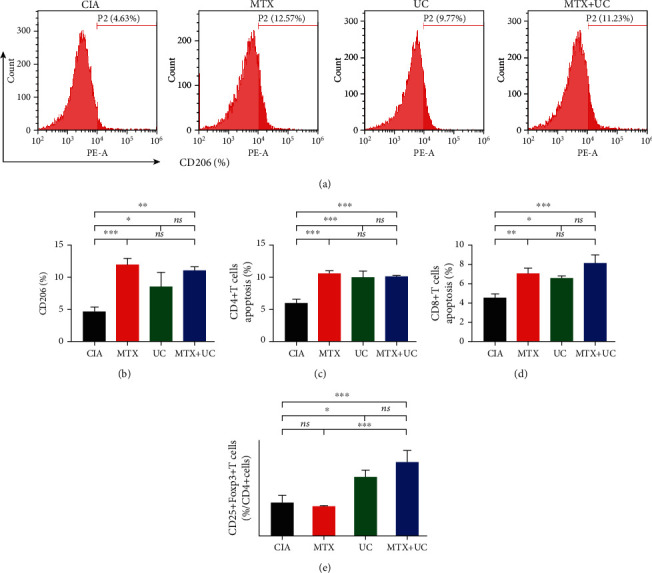
Combination of UCs and MTX promoted peritoneal macrophage polarization and enhanced the population of spleen Tregs in CIA mice. (a) Flow cytometry analysis of the proportion of M2 polarization in peritoneal macrophages marked by CD206. (b) The quantifications of CD206 macrophages after treatment. (c, d) The apoptosis of CD4+ and CD8+ spleen T cells was analyzed by flow cytometry. (e) The population of spleen Tregs marked by CD4+, CD25+, and Foxp3 was analyzed. *n* = 3 per group. Data are shown as mean ± SD. ^∗^*P* < 0.05; ^∗∗^*P* < 0.01; ^∗∗∗^*P* < 0.001; ns: not significant (*P* > 0.05).

It has been reported that both UCs and MTX play roles in immune regulation [20, 21]. Therefore, we evaluated whether the combined application of UCs and MTX in CIA mice could produce a synergistic effect on immunomodulation. M2 macrophages remove damaged tissues and pathogens leading to alleviated inflammation [22]. Treg cells, marked by CD4, CD25, and Foxp3 positive, are mainly responsible for suppressing the immune response and maintaining immune tolerance [23]. We observed that both MTX and UC treatment alone increased the CD206-positive M2 macrophages while combined application did not further promote M2 polarization of macrophages (Figures 4(a) and 4(b)). MTX or UC alone induced the apoptosis of CD4- and CD8-positive T cells, suggesting a suppressed inflammation response, and this effect cannot be strengthened by combined application of MTX and UCs (Figure S3a-d and Figures 4(c) and 4(d)). Moreover, UC alone could increase the population of Treg cells while MTX showed no effect on Treg proportion. Additionally, after combined MTX with UCs, the proportion of Tregs increased significantly compared with the MTX-treated group (Figure S3e and Figure 4(e)). In summary, the combined application of MTX and UCs showed better anti-inflammatory effects compared to MTX or UC alone treatment in CIA mice, which may be responsible for the better curative effect of combination medication.

### 3.6. MTX Has a Greater Effect on Facilitating M2 Macrophage Polarization than UCs

**Figure 5:**
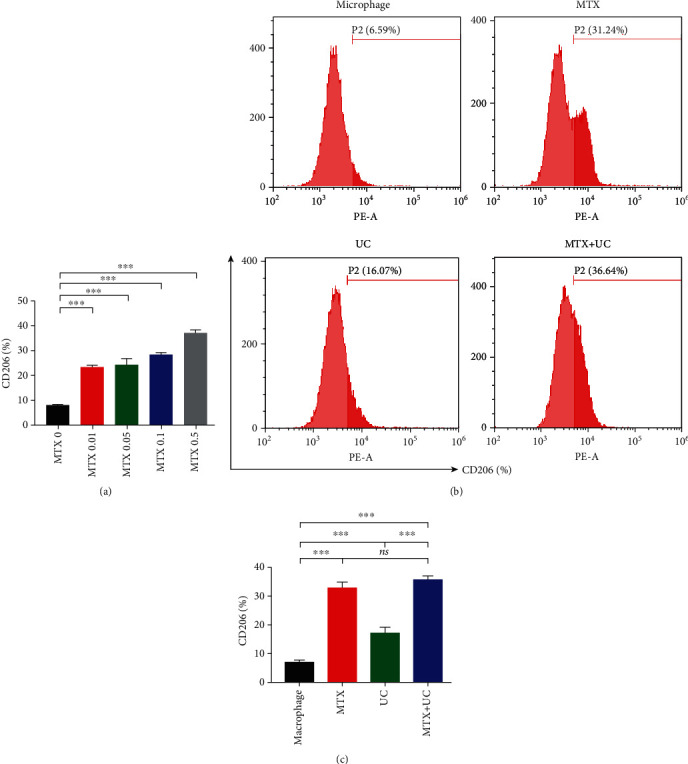
MTX prefers to facilitate macrophage polarization to M2 type. (a) MTX induced an increase of M2 macrophage polarization in a concentration-dependent manner in vitro. (b) The proportion of M2 phenotype macrophages in four groups was analyzed by flow cytometry. (c) The quantification showed that both MTX-treated macrophages and MTX+UC-treated macrophages showed more proportion of CD206-positive cells than macrophages cocultured with UCs. *n* = 3 per group. Data are shown as mean ± SD. ^∗^*P* < 0.05; ^∗∗^*P* < 0.01; ^∗∗∗^*P* < 0.001; ns: not significant (*P* > 0.05).

To further investigate the effects of MTX and UCs on macrophage polarization, we exposed Raw264.7 macrophages to MTX or UC treatment, or both of them. The results showed that MTX increased M2 macrophage polarization in a concentration-dependent manner (Figure 5(a)). UC alone also increased the M2 macrophages, but not as much as MTX (Figures 5(b) and 5(c)). In addition, the combined use of MTX and UCs increased the percentage of macrophage M2 polarization, which was similar to that of single MTX but higher than UC alone (Figures 5(b) and 5(c)). Those data indicated that MTX was more important in contributing to M2 macrophage polarization than UCs when used in combination.

## 4. Discussion

RA is a systemic autoimmune and inflammatory disease characterized by inflammation in the synovial membrane, which ultimately leads to joint destruction and deformity. MTX is a primary treatment for RA. It usually exerts a therapeutic effect at a low dose (15-25 mg weekly) [24]. However, a number of patients do not respond to MTX [4]. When this monotherapy is ineffective or adverse events emerge, other agents may be taken into consideration in conjunction with MTX for improved efficacy and safety. MSC-based therapy is a newly emerging strategy in recent years and has been evidenced to be a safe and effective method to alleviate RA symptoms. Therefore, MSCs have become a choice to collaborate with MTX. A previous study has revealed that the improvement of joint symptoms in CIA mice was more obvious when methotrexate and MSC transplantation were used [25]. Notably, in the present study, we started to inject MSCs after one week of MTX administration. This is similar to the remedy of clinical management. Here, we found that UCs and MTX combination can enhance the therapeutic effects and reduce adverse side effects induced by MTX alone.

MTX is a structural analogue of folic acid and blocks cell growth and reproduction via inhibiting dihydrofolate reductase [26]. It antagonizes purine synthesis and interferes with DNA synthesis, leading to cell cycle arrest in S-phase. Therefore, MTX prevents immune cell proliferation and suppresses the activation of the immune system, which is one of the main mechanisms of MTX to treat RA [2]. In addition to immunocytes, MTX also inhibits the proliferation of other cells, such as bone marrow cell, hepatocyte, pneumonocyte, nephrocyte, and gastrointestinal cells [6, 7, 27], leading to organic damage. Particularly, gastrointestinal toxicity is one of the most common side effects related to MTX, which results in malabsorption and diarrhea [28, 29]. In our study, we observed that MTX alone induced activated inflammation response in intestinal sections and disturbed the normal architecture of intestinal epithelium. The disrupted intestinal barrier leads to intestinal LPS translocation into circulation [30]. Therefore, the gastrointestinal toxicity of MTX could result in loss of body weight ever increased mortality [31]. MSCs have been thought of as an effective method to alleviate different types of small intestinal injuries [32–34]. Injection of BMMSCs could reduce colitis in mice via the release of TSG 6 independently of their localization to the intestine [35]. Besides, exosomes from MSCs have protective effects on colitis [36]. In our study, we found that intestinal disorders and increased mortality caused by MTX can be rescued by combined usage of UCs. Since MTX treatment was initiated before the UCs were infused, the intestinal epithelium might be already damaged by MTX [29]. The effect of UCs on restoring the damaged epithelium might be one of the mechanisms to intestinal recovery. Furthermore, it is notable that we also explored the effect of UCs on MODE-K cells when combined with MTX. We cultured MODE-K cells with MTX and UCs at the same time and found that the combination of MTX and UCs prevented impairment of vitality and proliferation on MODE-K cells by MTX treatment, which demonstrated that UCs could prevent gastrointestinal epithelium toxicity caused by MTX treatment. Those results strongly indicated that the combination of UCs and MTX improved the safety of MTX treatment.

The occurrence and development of RA disease are mediated by proinflammatory cytokines [37]. TNF-*α* and IL-1*β* accumulation in joint cavities contributes to synovial hyperplasia. It has been reported that macrophages and T cells are involved in regulating inflammatory response and the release of TNF-*α* and IL-1*β* in the progression of RA [22, 38]. Widely distributed activated macrophages in synovia are a hallmark of RA [39, 40]. There are two subtypes of macrophages existing as M1 and M2 macrophages. M1 phenotype macrophages, marked by CD68 and CD86, secrete proinflammatory factors and boost inflammatory response [22]. As a result, M1 phenotype polarization indicates the aggravation of inflammation. On the other hand, anti-inflammatory factors, such as IL-10 and TGF-*β*1, are secreted by CD206-marked M2 phenotype macrophages, which contribute to blocking inflammatory response and promoting tissue remodelling. In RA, the population of M1 is larger than that of M2 [41]. However, CD206-marked M2 macrophages produce anti-inflammatory factors contributing to block inflammatory response and promoting tissue remodelling. Recent studies have uncovered that the imbalance of M1 and M2 distribution is one of the principal causes of RA [22]. Researchers have illustrated that Notch, JNK, and ERK signalling pathways are involved in RA development for their disruption of M1/M2 equilibrium [41]. Therefore, manipulating the macrophage polarization could be a therapeutic target for RA. MTX could promote macrophage transition from M1 to M2 via adenosine A2a receptor, leading to inhibited cytokine expression and bone degradation [2]. In addition, it has been reported that MSCs also induce M2 polarization and exert anti-inflammatory effects [20, 42]. Here, we confirmed that both UCs and MTX induce macrophage polarization to M2 phenotype in RA, which may be the reason for reduced TNF-*α* and IL-1*β* in the synovial membrane. Intriguingly, we also found that MTX is more effective in inducing M2 macrophage polarization than UCs.

Besides macrophages, T cells also contribute a lot to the fluctuation of inflammatory cytokines [43–45]. CD4+ and CD8+ T cells are mainly responsible for boosting inflammation [45, 46]. On the other hand, Treg cells, marked by CD4+, CD25+, and Foxp3+, are mainly responsible for suppressing immune response and maintaining immune tolerance [16, 21, 47]. It has been reported that MTX can reduce the level of purine and pyridine pools in T cells and lead to reduction in T cell proliferation and increase in apoptosis [48]. The proportion of CD4+ T cells in the peripheral blood of patients with RA can be reduced by MTX treatment [49]. Moreover, MSCs also have been reported to suppress T cell activation and proliferation. In the present study, we observed both MTX and UC alone induced the apoptosis of CD4+ and CD8+ T cells. However, only UCs increased the population of Treg cells. Since the immune system is manipulated by the collaboration of different kinds of immune cells with various functions, it is the balance among these immune cells that counts for the homeostasis of the immune system and maintains the normal level of inflammation. In the present study, the observed increase in the frequency of Tregs among CD4+ cells may be a consequence of CD4+ T cell apoptosis. However, both MTX and UCs could increase the CD4+ T cell apoptosis to the same extent, but only UCs increased the population of Treg cells while MTX showed no effect on Treg proportion. Additionally, after combining MTX with UCs, the proportion of Tregs increased significantly compared with the MTX-treated group. Thus, the combined use of MTX and UCs could produce a stronger anti-inflammatory effect by the synergic action of reduced CD4+ and CD8+ T cells and increased the number of Treg cells.

## 5. Conclusions

In conclusion, our study reveals that combined application with UCs enhanced the safety and therapeutic efficacy of MTX. Therefore, the combined application of UCs and MTX should be seriously considered to be a new treatment for RA patients.

## Data Availability

The data used to support the findings of this study are available from the corresponding author upon request.

## Supplementary Materials

Supplementary Materials Supplementary Figure S1: the biologic characteristics of UCs have not been affected by MTX treatment. (a) Flow cytometric analysis of UCs revealed positive expression of CD90, CD73, and CD105 and negative expression of CD34 and CD45 with or without MTX treatment. (b) Representative images of Alizarin red staining of UCs after cultured in osteogenic inductive conditions for 28 days. (c) Representative images of Oil red O staining of UCs after cultured in adipogenic inductive conditions for 14 days. (d) MTT assay showed proliferation of UCs was not affected by MTX with different concentrations. (e) Apoptosis was analyzed by flow cytometry. Scale bar, 500 *μ*m. *n* = 3 per group. ns: not significant (*P* > 0.05). Supplementary Figure S2: combination of MTX and UCs rescued side effects of MTX. Histopathological examination of damages in the lungs, livers, and kidneys by H&E staining. MTX destroyed the organized architecture of the lungs, livers, and kidneys. And those adverse effects were mitigated by MTX and UC combination. Scale bar, 200 *μ*m. *n* = 6 per group. Figure S3: representative dot plots of flow cytometry analysis for CD4+ and CD8+ T cell apoptosis and Treg proportion. (a) Gating strategy of CD4+ T cells isolated from spleens of CIA mice. (b) Representative images of flow cytometry analysis for apoptotic CD4+ T cells. (c) Gating strategy of CD8+ T cells isolated from spleens of CIA mice. (d) Representative images of flow cytometry analysis for apoptotic CD8+ T cells. (e) Representative images of flow cytometry analysis for proportion of Tregs.
