# Supplementary material for: Mesenchymal Stem Cells Enhance Therapeutic Effect and Prevent Adverse Gastrointestinal Reaction of Methotrexate Treatment in Collagen-Induced Arthritis
Source: Stem Cells Int. 2021 Jan 9;2021:8850820. doi: 10.1155/2021/8850820 (PMC7814936; doi:10.1155/2021/8850820)
Supplement: Supplementary Materials — Supplementary Figure S1: the biologic characteristics of UCs have not been affected by MTX treatment. (a) Flow cytometric analysis of UCs revealed positive expression of CD90, CD73, and CD105 and negative expression of CD34 and CD45 with or without MTX treatment. (b) Representative images of Alizarin red staining of UCs after cultured in osteogenic inductive conditions for 28 days. (c) Representative images of Oil red O staining of UCs after cultured in adipogenic inductive conditions for 14 days. (d) MTT assay showed proliferation of UCs was not affected by MTX with different concentrations. (e) Apoptosis was analyzed by flow cytometry. Scale bar, 500 μm. n = 3 per group. ns: not significant (P > 0.05). Supplementary Figure S2: combination of MTX and UCs rescued side effects of MTX. Histopathological examination of damages in the lungs, livers, and kidneys by H&E staining. MTX destroyed the organized architecture of the lungs, livers, and kidneys. And those adverse effects were mitigated by MTX and UC combination. Scale bar, 200 μm. n = 6 per group. Figure S3: representative dot plots of flow cytometry analysis for CD4+ and CD8+ T cell apoptosis and Treg proportion. (a) Gating strategy of CD4+ T cells isolated from spleens of CIA mice. (b) Representative images of flow cytometry analysis for apoptotic CD4+ T cells. (c) Gating strategy of CD8+ T cells isolated from spleens of CIA mice. (d) Representative images of flow cytometry analysis for apoptotic CD8+ T cells. (e) Representative images of flow cytometry analysis for proportion of Tregs. [file 8850820.f1.pdf]

Supplementary Materials

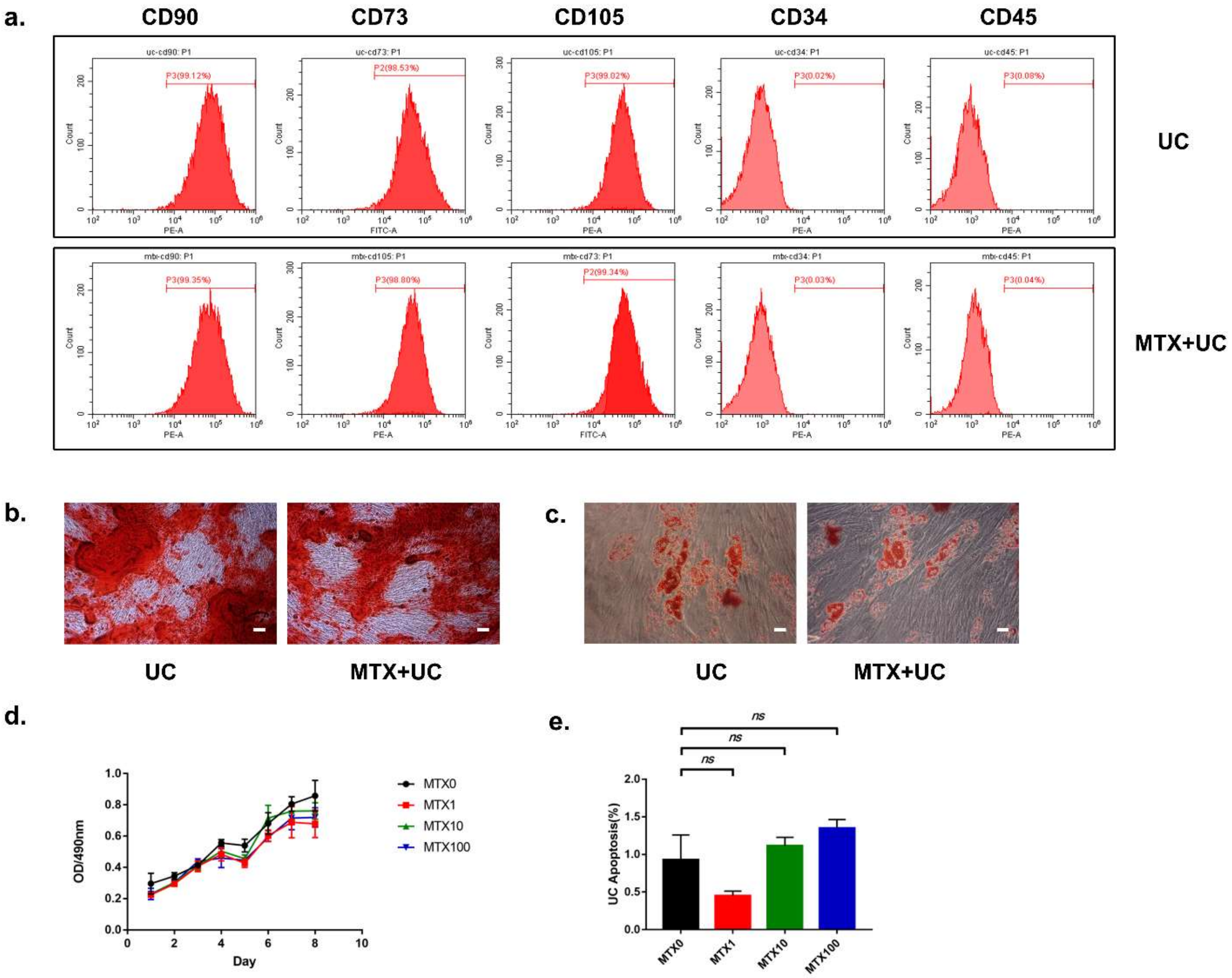

Figure S1. The biologic characteristics of UCs have not been affected by MTX treatment. (a) Flow cytometric analysis of UCs revealed positive expression of CD90, CD73, CD105, and negative expression of CD34, CD45 with or without MTX treatment. **(b)** Representative images of Alizarin Red staining of UCs after cultured in osteogenic inductive conditions for 28 days. **(c)** Representative images of Oil red O staining of UCs after cultured in adipogenic inductive conditions for 14 days. **(d)** MTT assay showed proliferation of UCs was not affected by MTX with different concentrations. **(e)** Apoptosis was analyzed by flow cytometry. Scale bar, 500μm. n=3 per group. ns, not significant (P>0.05).

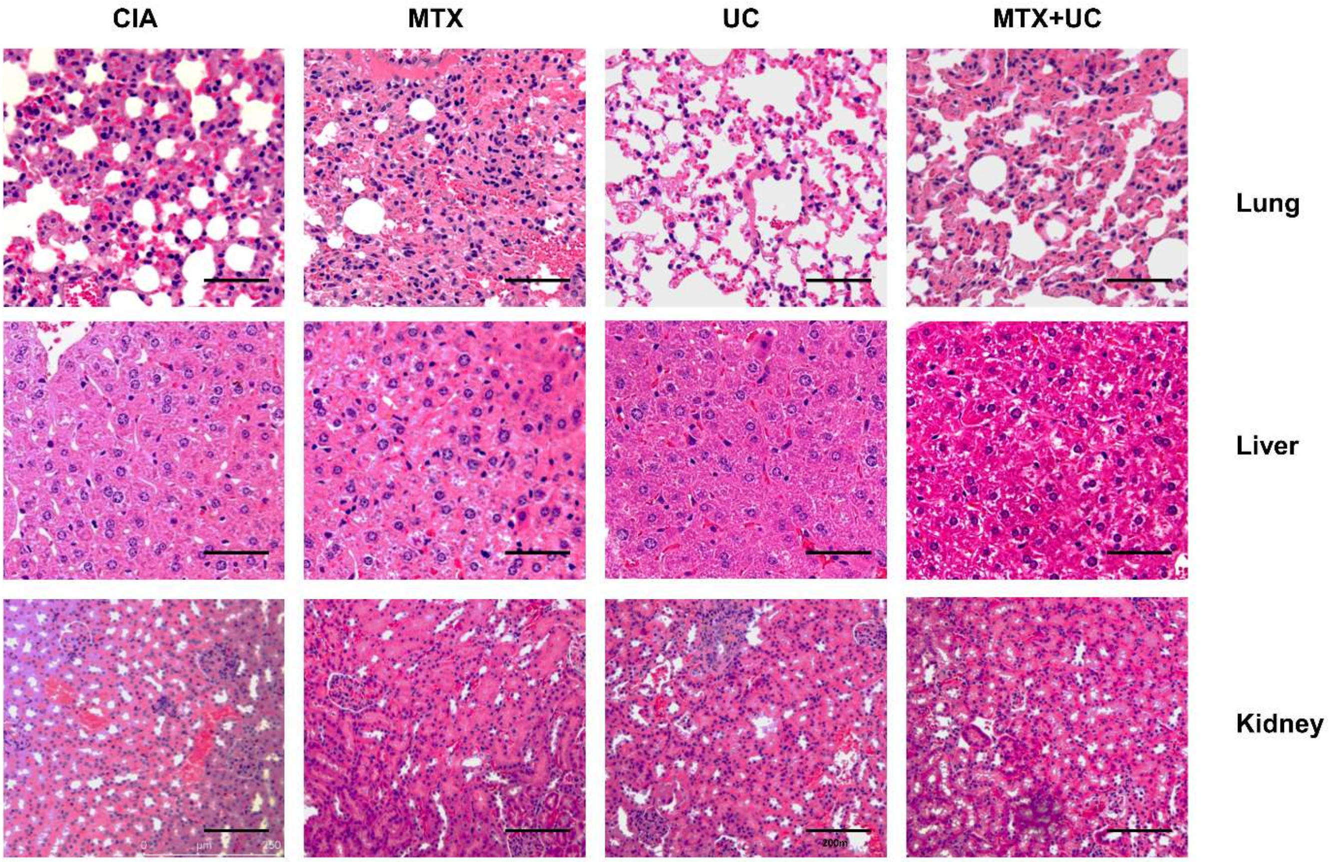

Figure S2. Combination of MTX and UCs rescued side effects of MTX. Histopathological examination of damages in lungs, livers and kidneys by H&E staining. MTX destroyed the organized architecture of lungs, livers and kidneys. And those adverse effects were mitigated by MTX and UCs combination. Scale bar, 200μm. n=6 per group.

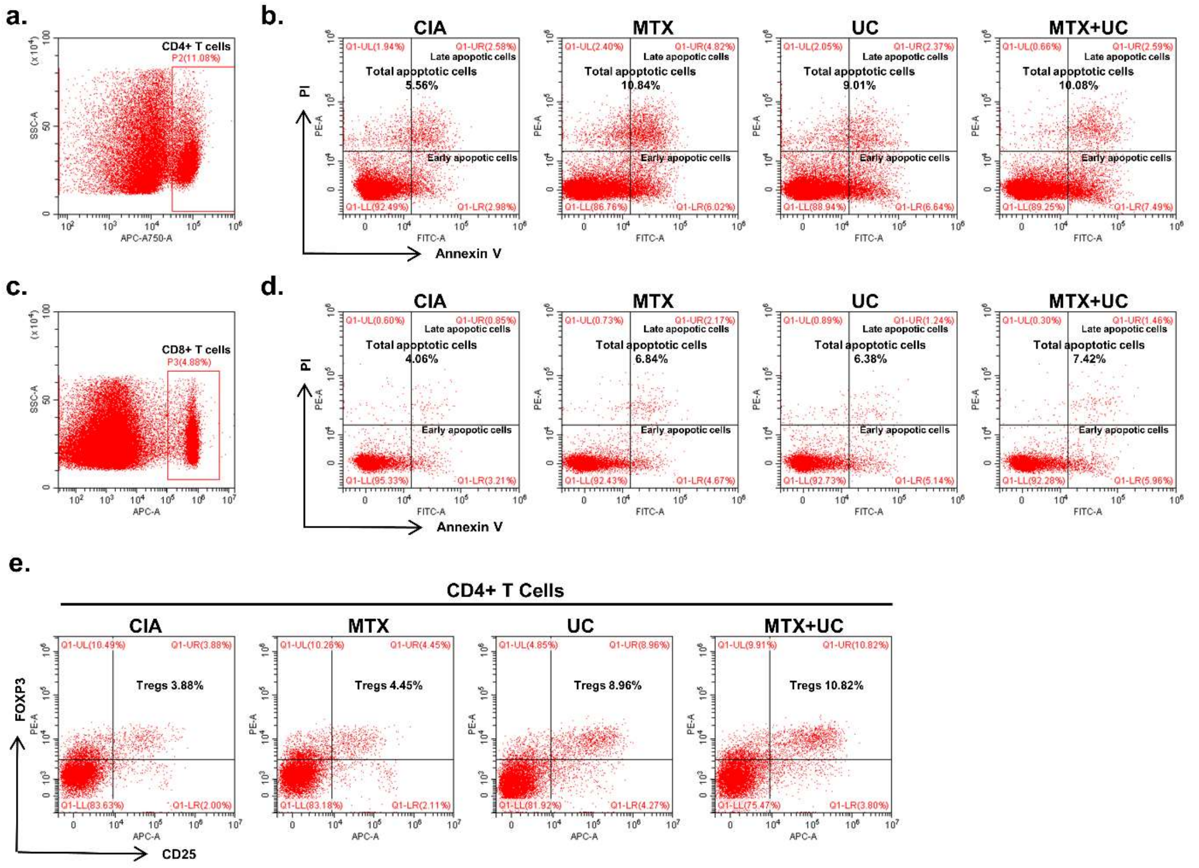

Figure S3. Representative dot-plots of flow cytometry analysis for CD4+ and CD8+ T cells apoptosis and Tregs proportion. **(a)** Gating strategy of CD4+ T cells isolated from spleens of CIA mice. **(b)** Representative images of flow cytometry analysis for apoptotic CD4+ T cells. **(c)** Gating strategy of CD8+ T cells isolated from spleens of CIA mice. **(d)** Representative images of flow cytometry analysis for apoptotic CD8+ T cells. **(e)** Representative images of flow cytometry analysis for proportion of Tregs.
